# A Silibinin‐Poly(ε‐Caprolactone) Conjugate as an Enhanced Anticancer Agent

**DOI:** 10.1002/mabi.202400510

**Published:** 2025-02-12

**Authors:** Fabiana Vento, Anna Privitera, Giuseppe Caruso, Angelo Nicosia

**Affiliations:** ^1^ Department of Chemical Sciences and INSTM UdR of Catania University of Catania V.le A. Doria 6 Catania 95125 Italy; ^2^ Department of Drug and Health Sciences University of Catania V.le A. Doria 6 Catania 95125 Italy; ^3^ Department of Biomedical and Biotechnological Sciences University of Catania Via Santa Sofia 97 Catania 95123 Italy; ^4^ Unit of Neuropharmacology and Translational Neurosciences Oasi Research Institute‐IRCCS Via Conte Ruggero 73 Troina 94018 Italy

**Keywords:** anticancer activity, facile synthesis, human colorectal adenocarcinoma, poly(ε‐caprolactone), silibinin

## Abstract

Poly(ε‐caprolactone) (PCL) is a hydrolytically degradable biopolyester used in drug delivery to enhance drug solubility and bioavailability, where drugs are typically incorporated physically within the biopolymeric matrix rather than covalently bonded, due to the limited availability of functional groups required for covalent attachment. In pursuit of developing a facile method for the production of a biopolyester‐drug covalent conjugate with effective drug loading capacity, this study reports the synthesis of a covalent Silibinin‐PCL conjugate (Sil‐PCL_Hyd_) through a two‐step approach. This involves the controlled hydrolysis of a high molecular weight PCL to increase the concentration of carboxylic end groups, which are subsequently used for the catalyzed esterification with Silibinin. The Sil‐PCL_Hyd_ is characterized with mass spectrometry, gel permeation chromatography, thermogravimetric analysis, differential scanning calorimetry, and NMR and UV–vis spectroscopies. The cytotoxic effects of Sil‐PCL_Hyd_ against colorectal adenocarcinoma cells (Caco‐2) are measured through the MTT assay. The results of the Sil‐PCL_Hyd_ characterization revealed a Silibinin loading of ≈9.8 wt.%. The MTT assay demonstrated that Sil‐PCL_Hyd_ induced cytotoxic effects at concentrations a hundred times lower than those required for free Silibinin. The proposed approach might represent a reliable pathway for the development of biopolyester‐based covalent conjugates with a high drug loading capacity.

## Graphical abstract

Poly(ε‐caprolactone) is used to synthesize a covalent Silibinin‐PCL conjugate. A two‐step method involving the hydrolysis of the PCL chain and its catalyzed esterification with Silibinin. The polymer conjugate showed significant cytotoxicity against colorectal adenocarcinoma cells at concentrations lower than those used for free Silibinin.

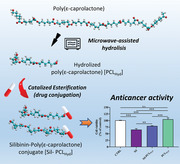

## 1 Introduction

In recent years, the development of novel drugs has been significantly inspired by nature,^[^ ^1^ ^]^ not only looking to traditional local medicine to found plant‐derived treatments for specific diseases,^[^ ^2^ ^]^ but also through the isolation of compounds and/or synthesis of novel drugs featuring molecular architectures derived from natural products.^[^ ^3^ ^]^ Moreover, well‐known and widely‐used anticancer drugs were originally sourced from natural origins, such as paclitaxel and others.^[^ ^4^ ^]^ Nevertheless, the derivation of drugs from natural sources does not guarantee their safety and efficacy in treatment, as the pharmacological active substances often exhibit poor solubility and bioavailability, necessitating the administration of high doses which may result in severe side effects.^[^ ^5^, ^6^ ^]^

Silibinin (Sil) constitutes the principal active compound within silymarin, a mixture of flavonolignans extracted from the seeds of *Silybum marianum*, commonly known as milk thistle plant.^[^ ^7^ ^]^ Recognized for its effectiveness in treating liver diseases, Sil has demonstrated a broad spectrum of biological effects, encompassing anticancer properties,^[^ ^8^, ^9^ ^]^ often ascribed to its capacity to induce apoptosis and suppress cell growth.^[^ ^10^, ^11^, ^12^ ^]^ Sil has exhibited significant cytotoxic effects against a range of cancer types, including prostate, bladder, lung, colon, and breast cancers.^[^ ^13^, ^14^, ^15^, ^16^, ^17^ ^]^ Moreover, it has been identified for its capacity to inhibit the invasiveness of cancer cells by mitigating the epithelial‐mesenchymal transition (EMT) in bladder transitional cell carcinoma.^[^ ^18^, ^19^ ^]^

Sil is typically administered via the oral route, and notwithstanding its demonstrated safety at high doses in numerous clinical trials, pharmacokinetic investigations have revealed that this drug exhibits poor absorption, limited oral bioavailability, and a short half‐life within the body,^[^ ^20^, ^21^, ^22^ ^]^ which is why developing new formulations to address these limitations is of significant interest.^[^ ^23^ ^]^

In this landscape, conjugation of bioactive molecules with polymer‐based carriers has demonstrated multiple advantages in drug delivery, including controlled release, enhanced efficacy, and reduced toxic response.^[^ ^24^, ^25^, ^26^, ^27^ ^]^

Innovative drug delivery systems based on natural and biodegradable polymers provide diverse opportunities to improve both the therapeutic efficacy and pharmacokinetic profiles of pharmaceuticals,^[^ ^28^, ^29^ ^]^ by also leveraging the distinctive attributes of the polymer matrices. Various biopolymers have been investigated for biomedical applications, ranging from alginate to silk fibroin.^[^ ^30^, ^31^ ^]^ Poly(ε‐caprolactone) (PCL) is a semicrystalline hydrophobic polymer characterized by a prolonged degradation period of up to 3–4 years, rendering it particularly suitable for tissue engineering applications.^[^ ^32^ ^]^ Moreover, its biocompatibility and biodegradability have prompted extensive research into its use as a carrier for controlled drug delivery.^[^ ^33^, ^34^, ^35^ ^]^ Numerous PCL‐drug formulations, which rely on the physical encapsulation of the drug within the polymer matrix through supramolecular approaches (e.g., nanosphere)^[^ ^36^ ^]^ have been proposed. These systems, leveraging their compatibility with a broad spectrum of drugs, facilitate homogenous drug distribution within the formulation matrix and allow drug release to occur over an extended period of several months.^[^ ^37^, ^38^ ^]^

Although physical inclusion might appear to be a straightforward method to load drugs onto polymer carriers, the design of polymer conjugates, which involves attaching the drug via a physiologically labile covalent bond to the polymer carrier, has established new frontiers in polymer‐based therapeutics.^[^ ^39^, ^40^ ^]^ Indeed, incorporating drugs physically into polymer particles may lead to a premature release under biological conditions, potentially occurring before the drug reaches its intended target site.^[^ ^41^ ^]^ On the other hand, covalent polymer‐drug conjugates allow for the release of the drug moiety solely through the cleavage of the labile bond initiated by external stimuli (e.g., pH variations and enzymatic cleavage), thereby conferring multiple advantages in terms of both the efficacy and pharmacokinetics of the drugs, while also facilitating the precise modulation of drug delivery.^[^ ^24^ ^]^ Within this framework, it is imperative that the polymer structure possesses appropriate functional groups that can be used for the attachment of the active drug.^[^ ^42^ ^]^ For instance, chitosan, a biopolysaccharide sourced from the exoskeletons of crustaceans, exhibits mucoadhesive properties that make it a suitable candidate for nose‐to‐brain drug delivery. The possibility of using its side‐chain amino groups for chemical functionalization facilitates the development of covalent conjugates based on chitosan.^[^ ^30^ ^]^ Furthermore, the hydroxyl groups present in polysaccharides can be used for this purpose. Specifically, in the case of dextran, complex synthetic pathways that involve the grafting of spermine onto the hydroxyl groups of dextran enable the development of drug delivery systems incorporating a variety of targeting agents.^[^ ^43^ ^]^ The combination of targeting molecules with species capable of molecular imaging has demonstrated promising outcomes in cancer diagnostics.^[^ ^44^, ^45^ ^]^

Biopolyesters possess only a single available functional group suitable for chemical functionalization, which has been used to conjugate targeting molecules in lieu of active pharmaceuticals. The latter are preferentially encapsulated within polymer nanoparticles to achieve adequate drug release efficiency.^[^ ^45^ ^]^

In the case of PCL, only the acidic end group is suitable for participation in the formation of ester or amide bonds with suitable pharmacologically active chemical species. Furthermore, given the high molecular weight of commercial PCL, which concomitantly presents a low concentration of end groups, it underscores the imperative to cut the PCL chain to reduce the molecular weight and enhance the concentration of end groups over weight.

To address this issue, the present study focused on the synthesis of covalent polymer‐drug conjugates by combining biodegradable PCL with Sil through a straightforward two‐step method, carefully designed to ensure an effective drug loading capacity. Specifically, the microwave‐assisted PCL hydrolysis was performed using *p*‐toluene sulfonic acid (pTSA) as the catalyst and water as the hydrolyzing agent. The resulting low molecular weight PCL (named PCL_Hyd_) was subsequently linked to the drug moiety through a Steglich catalyzed esterification reaction. The obtained Sil‐PCL_Hyd_ conjugate was characterized from a structural and chemical‐physical point of view through Gel Permeation Chromatography (GPC), Matrix‐Assisted Laser Desorption/Ionization – Time of Flight (MALDI‐TOF) mass spectrometry, NMR and UV‐Vis spectroscopies, as well as thermogravimetric analysis (TGA) and Differential Scanning Calorimetry (DSC). In vitro experiments employing human colorectal adenocarcinoma (Caco‐2) cells demonstrated enhanced anticancer activity of the Sil‐PCL_Hyd_ conjugate, exerting cytotoxic effects at a concentration one hundred times lower than that of free Sil. Consequently, the proposed approach may represent an innovative pathway for the development of more effective antineoplastic macromolecular systems, overcoming the challenges associated with the high molecular weight of conventional biopolyesters.

## 2 Results

### 2.1 Synthesis of the Silibinin‐Hydrolyzed Poly(ε‐Caprolactone) Conjugate

**Figure 1:**
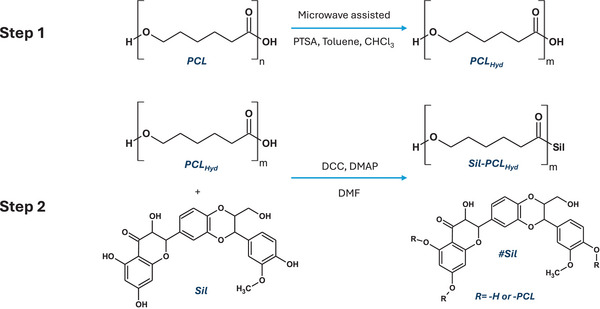
Schematic representation of the PCL conjugate synthesis approach.

To produce a covalent conjugate with Sil and PCL, it is imperative that the polymer structure, which provides only a single functional group suitable for drug linkage, is designed to enhance the concentration of the end groups. Consequently, the development of the Sil‐PCL_Hyd_ conjugate was performed through a two‐step procedure (**Figure** **1**) entailing: i) reduction of the molecular weight of PCL via microwave‐assisted hydrolysis; ii) a catalyzed esterification reaction to covalently attach the Sil molecule to the carboxylic end group of PCL_Hyd_.

Due to the high molecular weight of commercial PCL, hydrolysis is essential to enhance the concentration of carboxylic end groups for subsequent esterification with Sil.

In order to perform microwave‐assisted hydrolysis, PCL flakes were dissolved in a toluene:chloroform (4:1) mixture, with the addition of pTSA and a minimal amount of water, and were maintained in the microwave oven at reflux for 4 h. Finally, the conjugate Sil‐PCL_Hyd_ was obtained by Steglich esterification between PCL_Hyd_ and Sil, using N,N′‐Dicyclohexylcarbodiimide (DCC) and 4‐dimethylaminopyridine (DMAP) as coupling agents.^[^ ^25^, ^46^ ^]^

**Figure 2:**
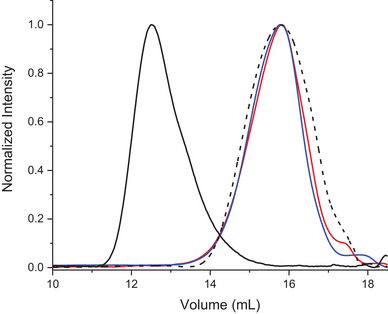
GPC curves of PCL (dRI detector, black line), PCL_Hyd_ (dRI detector, black dashed line) and Sil‐PCL_Hyd_ (dRI detector, blue line; UV–vis @287 nm, red line).

The average molecular weights of PCL and its derivatives were determined by GPC analysis. The commercial PCL exhibited a distribution curve (**Figure** **2**, black line) centered at ≈12.5 mL, with a $\bar{M_{w}}$ of 72 KDa (PDI = 1.4). As a consequence of microwave‐assisted hydrolysis, the GPC curve of PCL_Hyd_ (Figure 2, black dashed line) shifted to a higher elution volume (≈15.8 mL), with a $\bar{M_{w}}$ of 6.4 KDa (PDI = 1.3). The polymerization degree resulting from the obtained $\bar{M_{n}}$ allowed for the quantitative estimation of the carboxylic groups potentially present in each sample. PCL showed ≈0.2% of carboxylic groups in relation to the number of repeating units. As a result of microwave‐assisted hydrolysis, PCL_Hyd_ exhibited an increased concentration of acid groups, ≈2.4%.

The effective binding of Sil to PCL_Hyd_ was validated through the GPC analysis of Sil‐PCL_Hyd_, using both the differential refractive index (dRI) and the UV–vis spectrophotometer (set at the absorption wavelength of Sil, λ = 330 nm) as detectors. The overlap of the chromatograms obtained from the two detectors confirmed the covalent attachment of Sil to the PCL_Hyd_ chain (Figure 2, red and blue continuous lines). The $\bar{M_{w}}$ of Sil‐PCL_Hyd_, determined with GPC analyses, was 6.9 KDa with a PDI of ≈1.1. The observed increase of $\bar{M_{w}}$ is attributed to the attachment of the Sil moiety as end group of the PCL_Hyd_ chain, while the decrease in PDI can be ascribed to the purification procedure performed subsequent to the esterification reaction, which results in the elimination of low molecular weight oligomers.

**Figure 3:**
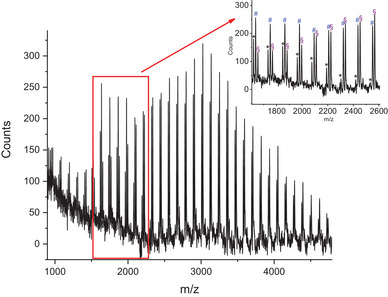
MALDI‐TOF mass spectrum of the PCL_Hyd_.

The structural analysis of PCL_Hyd_ and its conjugate was further pursued with mass spectrometry. The MALDI‐TOF mass spectrum of PCL_Hyd_ (**Figure** **3**) shows the expected peaks attributed to the [H‐[M]_n_‐OH] Na^+^ and [H‐[M]_n_‐OH] K^+^ species, respectively, at m/z 1067.8 + *n*114 (#) and 1083.8 + *n*114 (§) (*n* = 0‐33). Additionally, the spectrum shows the presence of low intensity signals attributable to the cyclic species c‐[M]_n_ (*n* = 0–14), detected at 1049.8 + *n*114 (*) as an adduct with Na^+^. For the sake of clarity, the formulas indicate [M] as the caprolactone repeating unit (CL), 114 as the molecular mass of the CL repeating unit, and *n* as the number of CL units to be added to the individual species. As an example, the signal at 1049.8 is the first detectable signal in the mass spectrum, corresponding to the c‐[M]_n_ species with a polymerization degree of 9, detected as an adduct with sodium cation; the signal at 1067.8 pertains to the first detected open‐chain species [H‐[M]_n_‐OH] Na^+^ with a polymerization degree of 9, and the signal at 1083.8 corresponds to the same species detected as an adduct with potassium cation.

**Figure 4:**
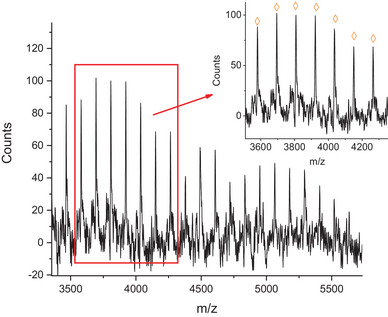
MALDI‐TOF mass spectrum of Sil‐PCL_Hyd_.

On the other hand, the MALDI‐TOF mass spectrum of Sil‐PCL_Hyd_ (**Figure** **4**) shows a series of peaks at m/z values 3465.9 + *n*114 (*n* = 0–19), detected as [Sil‐[M]_n_‐OH]Na^+^ adducts, thereby confirming the complete functionalization with Sil of the acid end group of PCL_Hyd_. The absence of additional peaks attributable to cyclic species may be due to the adopted purification procedure.

**Figure 5:**
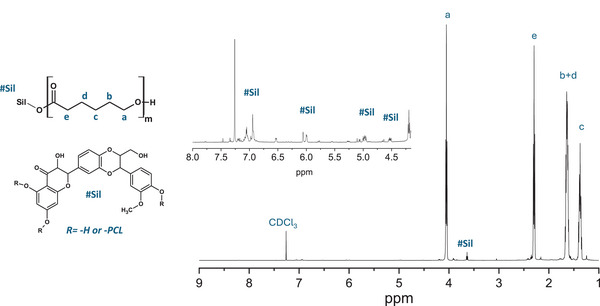
^1^H‐NMR analysis of the Sil‐PCL_Hyd_ conjugate.

The ^1^H‐NMR spectrum of the Sil‐PCL_Hyd_ conjugate is shown in **Figure** **5**. The peaks corresponding to the PCL chain are observed at 4.06 ppm (triplet, 2H, a), 2.31 ppm (triplet, 2H, e), 1.65 ppm (multiplet, 4H, b + d), and 1.38 ppm (quintuplet, 2H, c). Considering the integrations of the peaks associated with the flavanol and aromatic groups of the Sil molecule^[^ ^47^ ^]^ (4.5 – 7.1 ppm, 0.25 H, #Sil) with those of PCL_Hyd_, it was determined that the loading of Sil on PCL was ≈2.5 mol% (9.8 wt.%).

**Figure 6:**
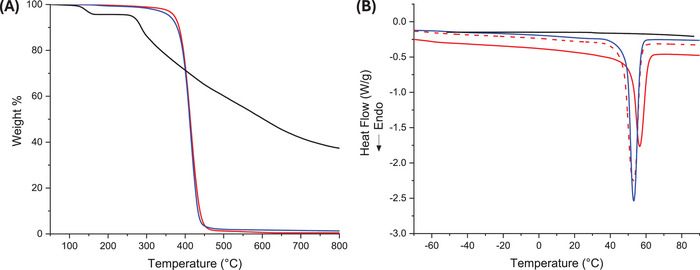
A) TGA and B) DSC traces of Sil (black solid line), PCL (red solid line), PCL_Hyd_ (red dashed line), and Sil‐PCL_Hyd_ (blue solid line).

The thermal properties of the polymers were investigated by TGA and DSC analyses (**Figure** **6**). In particular, TGA measurement shows that Sil (Figure 6A) undergoes a primary thermal degradation step at ≈126 °C (T_onset_), followed by a second step at 270 °C, and a continuous weight loss that ends at 800 °C with a residue of ≈37 wt.%. PCL shows a single‐step degradation at ≈395 °C (T_onset_), which culminates in a final residue of 0.5 wt.%, where the degradation process is composed of two concurrent mechanisms: the cis‐elimination reaction and chain end scission.^[^ ^48^, ^49^ ^]^ The Sil‐PCL_Hyd_ exhibits a thermogram that closely resemble that of PCL, yet it is characterized by higher residue at 800 °C, measuring ≈1.3 wt.%. The DSC analysis (Figure 6B) indicates the PCL melting temperature at ≈56.5 °C (ΔH = 56.9 J/g), aligning with the findings reported in the literature,^[^ ^50^ ^]^ while PCL_Hyd_ presents a lower melting temperature at 52.7 °C (ΔH = 85.0 J/g). This reduction is attributed to the decrease in the average molecular weight of PCL^[^ ^50^, ^51^ ^]^ from $\bar{M_{w}}$ 72 KDa to 6.4 KDa. Notably, the increase in the melting enthalpy for PCL_Hyd_ signifies a higher crystallinity than that of PCL, likely resulting from a decrease in polymer entanglement density and increased chain diffusion.^[^ ^50^ ^]^

Finally, as expected, the DSC trace of Sil‐PCL_Hyd_ shows a behavior closely akin to that of PCL_Hyd_, with the melting temperature recorded at 53.0 °C (ΔH = 82.12 J/g, determined by evaluating only the PCL content). The presence of the Sil moiety as a terminal group of the polymer chains slightly affects the crystallinity of the system, as evidenced by the observed reduction in melting enthalpy.

### 2.2 Cytotoxic Activity in Caco‐2 Cells

This set of experiments was carried out to investigate the toxic effects exerted by Sil‐PCL_Hyd_ conjugates on Caco‐2 cells and compare it with that observed using the free form of the drug (Sil).

**Figure 7:**
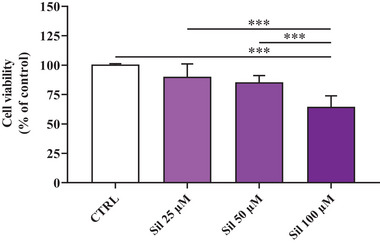
Cell viability analysis assessed by the MTT assay. Caco‐2 cells were left untreated (CTRL) or treated with increasing concentrations (25, 50, or 100 µm) of Sil for 24 h. Data are the mean of six to ten values and are expressed as the percent variation with respect to the cell viability recorded in CTRL cells. Standard deviations are represented by vertical bars. Significantly different, ***p < 0.001.

The primary objective was to evaluate the toxic effects of increasing concentrations (25, 50, or 100 µm) of Sil on Caco‐2 cells after a 24 h treatment period. As reported in **Figure** **7**, 100 µm was identified as the minimum concentration of Sil capable of inducing significant toxic effects on tumor cells compared to untreated cells, while only a tendency toward the decrease of cell viability was observed with 25 or 50 µm concentrations.

**Figure 8:**
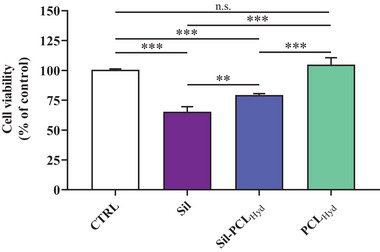
Cell viability analysis was assessed by MTT assay. Caco‐2 cells were left untreated (CTRL) or treated with Sil (100 µm), Sil‐PCL_Hyd_ (0.9 µm in Sil, 0.6 µm in PCL_Hyd_) or PCL_Hyd_ (0.6 µm) for 24 h. Data are the mean of three to four values and are expressed as the percentage variation with respect to the cell viability recorded in CTRL cells. Standard deviations are represented by vertical bars. Significantly different, **p < 0.01; ***p < 0.001.

Subsequent analysis involved the evaluation and comparison of the toxic activity of Sil and the Sil‐PCL_Hyd_ conjugate. **Figure** **8** illustrates the comparison between Sil at a concentration of 100 µm, and the Sil‐PCL_Hyd_ conjugate at a concentration of 0.9 µm in Sil.

The experimental findings not only confirmed the toxicity induced by Sil at 100 µm but also demonstrated the ability of the Sil‐PCL_Hyd_ conjugate to significantly decrease the viability of Caco‐2 cells. This result is particularly interesting considering that the Sil‐PCL_Hyd_ conjugates comprise only 9.8 wt.% of the drug, indicating that Sil exerts a toxic potential at concentrations approximately one hundred times lower when attached to the conjugate. Furthermore, the fact that PCL used alone did not exert any toxic effects provides evidence that the observed toxicity when using Sil‐PCL_Hyd_ conjugates is attributable solely to the drug, rather than to the presence of the PCL_Hyd_ vehicle. Higher concentrations of the Sil‐PCL_Hyd_ conjugate were excluded from consideration due to the toxic effects exerted by the vehicle per se.

These findings corroborates previous preclinical studies showing the efficacy of Sil in promoting the citotoxicity in gastric cancer (BGC823, SNU216, SNU668, and SGC7901 cells),^[^ ^52^, ^53^, ^54^ ^]^ laryngeal squamous cell carcinoma (Hep‐2 cells),^[^ ^55^ ^]^ gastric adenocarcinoma (AGS cells),^[^ ^56^ ^]^ and ovarian cancer.^[^ ^57^ ^]^ Furthermore, those studies also indicate the capability of PCL to improve the accumulation and cytotoxic activity of drugs against Caco‐2 cells^[^ ^58^, ^59^ ^]^ and various other cancerous cell lines,^[^ ^60^, ^61^ ^]^ emphasizing the promising role of PCL as a vehicle for the delivery of anticancer drugs.

## 3 Conclusion

A new PCL‐based drug delivery system containing Sil (Sil‐PCL_Hyd_ conjugate) was synthesized and characterized. The typical challenge associated with high‐molecular weight biopolyesters, which possess a low concentration of functional groups for covalent functionalization with drug moieties, was addressed using a two‐step method. This involved i) microwave‐assisted hydrolysis of PCL to reduce the average molecular weight and increase the concentration of end groups, and ii) a catalyzed esterification reaction to covalently link the polymer chain with Sil. The characterization of the polymer‐drug conjugate confirmed the successful covalent functionalization of the end groups of the polymer with Sil, resulting in a drug loading of about 9.8 %wt.

The in vitro experiments demonstrated the enhanced anticancer activity of the Sil‐PCL_Hyd_ conjugate compared to free Sil against Caco‐2 cells, which represent a well‐known experimental model of solid tumors.^[^ ^62^, ^63^, ^64^ ^]^ The comparison between the toxic activity of free Sil and that exerted by the Sil‐PCL_Hyd_ conjugate allowed us to demonstrate the ability of our system to significantly decrease the viability of Caco‐2 cells at a significantly lower concentration (0.9 µm) compared to the free drug (100 µm). This phenomenon could be attributed to the controlled release of the drug as a result of hydrolysis of the drug‐linked ester bond in the cellular environment.

The findings reported here are of particular relevance when evaluating the potential adverse effects associated with the use of milk thistle or Sil, which include headaches, neuropsychological events (e.g., asthenia, malaise, and insomnia), gastroenteritis, and anaphylactic reactions.^[^ ^65^, ^66^, ^67^, ^68^, ^69^ ^]^ Various studies investigating the antitumoral activity of Sil in vivo typically use dose ranges of 100 to 300 mg Kg^−1^.^[^ ^70^, ^71^, ^72^, ^73^ ^]^ Considering that Sil exhibits activity at a concentration approximately one hundred times lower when conjugated with PCL, the dosage of the Sil‐PCL_Hyd_ conjugate to be administered in vivo could potentially be significantly lower, thus presenting the opportunity to enhance the cytotoxic effects of the drug while simultaneously mitigating the incidence of possible adverse effects.

## 4 Experimental Section

### Materials

PCL, pTSA, toluene, chloroform, deuterated chloroform (CDCl_3_), methanol, tetrahydrofuran (THF), anhydrous N,N‐dimetilformamide (DMF), Dimethylsulfoxide (DMSO), Sil, DMAP, DCC, *trans*‐2‐[3‐(4‐tert‐butylphenyl)‐2‐methyl‐2‐propenylidene]‐malononitrile (DCTB), Dulbecco's Modified Eagle Medium (DMEM), penicillin, streptomycin, 3‐(4,5‐Dimethylthiazol‐2‐yl)‐2,5‐Diphenyltetrazolium Bromide (MTT), dimethyl sulfoxide (DMSO), and GlutaMAX™ were supplied by Sigma–Aldrich (Merck Life Science S.r.l., Milan, Italy). Human Caco‐2 cells (ATCC HTB‐37^TM^), trypsin‐EDTA solution, and fetal bovine serum (FBS), were purchased from American Type Culture Collection (ATCC, Manassas, VA, USA). C‐Chip disposable hemocytometers were obtained from Li StarFish S.r.l. (Naviglio, MI, Italy). All reagents and chemicals used in the present study were analytical grade.

### Instrumentation

UV–vis spectra were recorded using an Agilent Cary60 UV–vis Spectrophotometer, using quartz cuvettes with a path length of 1 cm and THF as solvent, at 25 ± 0.1 °C.

TGA were performed using a Perkin‐Elmer TGA 7 equipped with a TAC 7/DX with a thermal ramp of 10 °C min^−1^, in an inert atmosphere (N_2_).

DSC measurements were performed using a TA Q20 instrument (TA Instruments), equipped with a refrigerant cooling system (RCS 90, TA Instruments), with a heating rate of 10 °C min^−1^, in the specific temperature range and in an anhydrous N_2_ atmosphere (60 mL min^−1^).

MALDI‐TOF mass spectra were acquired by a Voyager DE (PerSeptive Biosystem) using an acquisition protocol reported elsewhere.^[^ ^74^, ^75^, ^76^ ^]^ The calibration of the MALDI‐TOF mass spectrometer and the determination of the average molecular mass were performed as reported in previous cases.^[^ ^77^, ^78^, ^79^, ^80^ ^]^ The DCTB was used as a matrix.

^1^H NMR spectra were acquired on a ^UNITY−^INOVA Varian instrument operating at 500 MHz, using VNMR for software acquisition and processing, using CDCl_3_ as solvent. Chemical shifts were expressed in ppm.

GPC experiments were performed using a PL‐GPC 110 (Polymer Laboratories) thermostated system, equipped with three PL‐gel 5 mm columns (two Mixed‐D and one Mixed‐E) joined in series. A UV–vis spectrophotometer (Hewlett Packard series 1050) connected in series with a DAWN multiangle laser light scattering (Wyatt Technology) detector, together connected in parallel with a dRI, were used as detectors. The analyses were performed at 35 ± 0.1 °C using THF as eluent at a flow rate of 1 mL min^−1^. The dn/dc value of PCL in THF was fixed at 0.053 mL g^−1^.^[^ ^81^ ^]^ The acquired data were analyzed using ASTRA 6.0.1.10 software (Wyatt Technology).

Dynamic Light Scattering (DLS) measurements were performed using a miniDAWN Treos (Wyatt Technology) multi‐angle laser light scattering detector, equipped with a Wyatt QELS DLS module, at 25 °C. The size distributions of the particles were analyzed using ASTRA 6.0.1.10 software (Wyatt Technology).

### Microwave‐Assisted Hydrolysis of Poly(ε‐Caprolactone)

The reduction of the molecular weight of PCL was achieved through catalyzed microwave‐assisted hydrolysis.^[^ ^82^ ^]^ Briefly, PCL (2 g, 0.02 mmol) having $\bar{M_{w}}$ = 72 KDa (Polydispersity Index, PDI = 1.4) was dissolved in a 4:1 toluene/chloroform mixture (50 mL) and poured into a round‐bottom flask. Subsequently, pTSA (500 mg, 2.90 mmol) and water (500 µL) were added to the reaction mixture. The microwave‐assisted hydrolysis was carried out in a single‐mode microwave reactor (CEM Discover S‐Class) under open‐vessel conditions. The reaction mixture was heated to 150 °C with 100 W power and stirred under reflux for 4 h, after which it was cooled to room temperature. The solid product, obtained following roto‐evaporation, was dissolved in a small volume of chloroform, purified through precipitation in water, and dried in a vacuum oven (50 °C, 24 h), yielding PCL_Hyd_ with a $\bar{M_{w}}$ of 6.4 KDa (PDI = 1.4).

### Synthesis of the Silibinin‐Hydrolyzed Poly(ε‐Caprolactone) Conjugate

The Sil‐PCL_Hyd_ was obtained via a Steglich esterification reaction between the PCL_Hyd_ homopolymer and Sil. Briefly, to a stirred solution of PCL_Hyd_ (100 mg, 16 µmol) and Sil (24 mg, 52 µmol) in anhydrous DMF (1 mL), DMAP (1 mg, 8 µmol) was added. The mixture was maintained at 0 °C for 15 min, following which DCC (3.5 mg, 19 µmol) was introduced. The reaction mixture was gradually brought to room temperature, and stirring was continued for an additional 100 h under anhydrous and inert conditions. Subsequently, the reaction mixture was centrifuged (9000 rpm, 15 min), and the supernatant was precipitated twice in cold methanol. The resulting solid product (Sil‐PCL_Hyd_) was dried in a vacuum oven (50 °C, 24 h).

### Cell Culture, Treatment Protocol, and Evaluation of Anticancer Activity

To evaluate the anticancer activity of the newly synthesized conjugates, Caco‐2 cells were employed as a model of solid tumor cells.^[^ ^62^, ^63^ ^]^ The cells were grown in DMEM enriched with heat‐inactivated FBS (10%), penicillin (50 units mL^−1^), streptomycin (50 µg mL^−1^), and GlutaMAX™ (2 mM), maintained in polystyrene cell culture flasks (25 or 75 cm^2^) in a humidified atmosphere (37 °C and 5% CO_2_), and passaged every 2–3 days based on cell confluence to prevent overgrowth.^[^ ^63^ ^]^

The day before treatment, cells were harvested, counted through a C‐Chip disposable hemocytometer, and seeded in 96‐well plates (1.5×10^4^ cells well^−1^). Stock solutions of Sil (free drug), Sil‐PCL_Hyd_ conjugates (containing 9.8 wt.% of the drug), or PCL were prepared in DMSO at a concentration of 2.4 mg mL^−1^ and subsequently diluted to the final required concentration in complete medium. The following day, cells were treated with Sil (25, 50, or 100 µm), Sil‐PCL_Hyd_ conjugate (0.9 µm in Sil, 0.6 µm in PCL_Hyd_) or PCL_Hyd_ (0.6 µm), followed by a 24 h incubation in a humidified environment. Upon completion of the treatment, the changes in cell viability of the Caco‐2 cells under these experimental conditions were assessed using the MTT assay, as previously detailed.^[^ ^63^ ^]^ Ultimately, absorbance at 569 nm was measured with a Synergy H1 Hybrid Multi‐Mode Microplate Reader (Biotek, Shoreline, WA, USA). Cell viability values were normalized with respect to that measured in untreated Caco‐2 cells (representing the controls).

The long‐term stability of the Sil‐PCL_Hyd_ conjugate was assessed through DLS measurements (data not shown). The pristine Sil‐PCL_Hyd_ in aqueous media exhibited two broad size distributions, having average hydrodynamic radii of 210 nm for the main distribution and 5 nm for the minor distribution, respectively. After a storage period of three days, minimal variations (<10%) were observed in the average hydrodynamic radius of the main distribution.

### Statistical Analysis

Statistical analysis was performed using Graphpad Prism software (V 8.0) (Graphpad software, San Diego, CA, USA). One‐way analysis of variance (ANOVA) followed by Tukey's *post hoc* test was used for multiple comparisons. Only two‐tailed p‐values less than 0.05 were considered statistically significant. Data were reported as mean ± SD.

## Conflict of Interest

The authors declare no conflict of interest.

## Author Contributions

F.V.: investigation, methodology, resources, formal analysis, data curation, writing ‐ original draft preparation. A.P.: formal analysis, investigation, writing ‐ original draft preparation, writing ‐ review and editing, visualization. G.C.: formal analysis, investigation, data curation, resources, writing ‐ original draft preparation, writing ‐ review and editing, visualization, funding acquisition. A.N.: conceptualization, methodology, validation, formal analysis, resources, data curation, writing ‐ original draft preparation, writing ‐ review and editing, visualization, supervision, project administration, funding acquisition.

## Data Availability

The data that support the findings of this study are available from the corresponding author upon reasonable request.
